# Supplementary material for: Low cost, high performance processing of single particle cryo-electron microscopy data in the cloud
Source: eLife. 2015 May 8;4:e06664. doi: 10.7554/eLife.06664 (PMC4440898; doi:10.7554/eLife.06664)
Supplement: Supplementary file 1. — Step-by-step tutorial describing how to use Amazon's EC2 environment to analyze cryo-EM data. DOI: http://dx.doi.org/10.7554/eLife.06664.010 [file elife06664s003.pdf]

Electron microscopy image analysis in the cloud

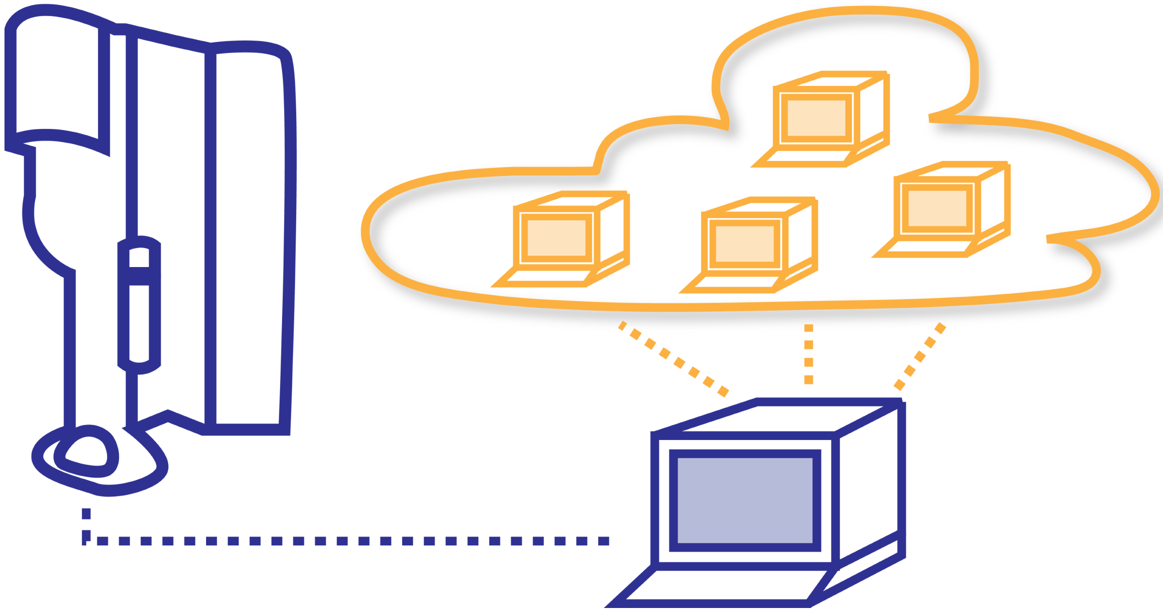

Low cost, high performance processing of single particle cryo-EM data in the cloud: Tutorial & workflow

Michael A. Cianfrocco & Andres E. Leschziner (2014)

Table of Contents

Getting started....3

Terminology 3

Amazon instance types....3

Pricing 4

Instance request limits 4

Setting up your account 4

Security considerations....4

Setting up a single machine....7

Launching a single instance with EM-packages-in-the-cloud 7

Spot instance requests 12

EM-Packages-in-the-Cloud AMI 12

Running commands....13

Data storage 13

Create a volume on the Amazon EC2 management console 13

Attaching a volume to an instance....14

Mounting a volume 14

Upload your data 15

Unmounting a volume....15

Setting up a cluster....15

Configuring your cluster through .starcluster/config 16

Gathering security credentials 16

Editing .starcluster/config....17

Create keypair 17

On-demand vs. spot instances 17

Starting a STARcluster....18

Logging into the cluster....18

Example job submission script 19

Useful cluster commands 19

Stopping cluster 20

Troubleshooting 20

Getting started

Terminology

Before you start, there is some terminology that will be helpful for you to know:

AWS (Amazon Web Services) The branch of Amazon that runs & administers to their cloud services.

EC2 (Elastic Compute Cloud) The name for Amazon's cloud computing infrastructure.

EBS (Elastic Block Storage) Persistent storage servers to which your data will be uploaded and backed up throughout your computations.

Instances The name given to individual computing nodes within Amazon's EC2. Amazon instances are the computing nodes that are grouped together into clusters by the Starcluster program.

Starcluster An open source toolkit that allows users on Amazon's EC2 to group instances together into a Sun Grid Engine cluster.

Key Pairs A security measure to encrypt and decrypt login information on Amazon's cloud infrastructure. Practically, this means that both you and Amazon have files that are used to log into an Amazon instance. The encryption key on your computer must be used when trying to ssh or scp files from the Amazon server, specified with the '-i' input command.

AMI (Amazon machine image) A template used by Amazon when starting an instance. It provides the instance with the operating system and applications that have saved previously as the AMI.

Amazon instance types

General purpose ('T2'):
- Baseline performance
- 1 - 2 CPUs
- 1 - 4 GB memory

General purpose ('M3'):
- Balances computing, memory, and network resources
- 1 - 8 CPUs
- 3.75 - 30 GB memory

Compute optimized (‘C3' & ‘C4’):
- High performance processors: Intel Xeon E5-2680 v2 (Ivy Bridge)
- Support for enhanced networking
- 2 - 32 CPUs
- 3.75 - 60 GB memory

Memory optimized ('R3'):
- High performance processors: Intel Xeon E5-2670 v2 (Ivy Bridge)
- Support for enhanced networking
- 2 - 32 CPUs
- 15 - 244 GB memory

GPU enabled ('G2'):
- High performance processors: Intel Xeon E5-2670 v2 (Ivy Bridge)
- High-performance NVIDIA GPU with 1,536 CUDA cores and 4GB of video memory
- Support for enhanced networking
- 8 CPUs
- 15 GB memory

You can read more here.

Pricing

Amazon prices their instances depending on the number of CPUs and the type of instance. You can consult the hourly rates of each instance here.

New users get free access to the entry level instances ('t2.micro') for 12 months.

Instance request limits

After creating an account, you can find out the instance request limits on the EC2 console screen under the tab 'Limits.'

Setting up your account

Before you can get access to the elastic computing cloud, you'll need to create an Amazon Web Services account. Even if you already have an Amazon account that you use for other services from Amazon, you'll still have to create an 'Amazon Web Services' account.

To sign up, go to the AWS website and click 'Sign Up': http://aws.amazon.com/

You can also watch a video tutorial that will walk you through making an account.

Note: Make sure you select 'Basic' plan (which is free).

Security considerations

Now that you've created your account, you should perform a few tasks that are recommended by Amazon Web Services to ensure that your account is secure. You should do these extra steps because the account that you just set up has root privileges which means that anyone

who logs in with these credentials can get credit card info, delete users, prevent access, etc. [Which is a scenario that definitely happens.]

Overall process
1. Set up multi-factor authentication for root access
2. Create new user & give this user administrative privileges
3. Set up multi-factor authentication for user
4. (Optional) Repeat for users from your lab

1. Set up multi-factor authentication for root access

Navigate to the IAM (Identity and Access Management) interface. Click on the top left menu icon '**Services**' and then select '**IAM**':

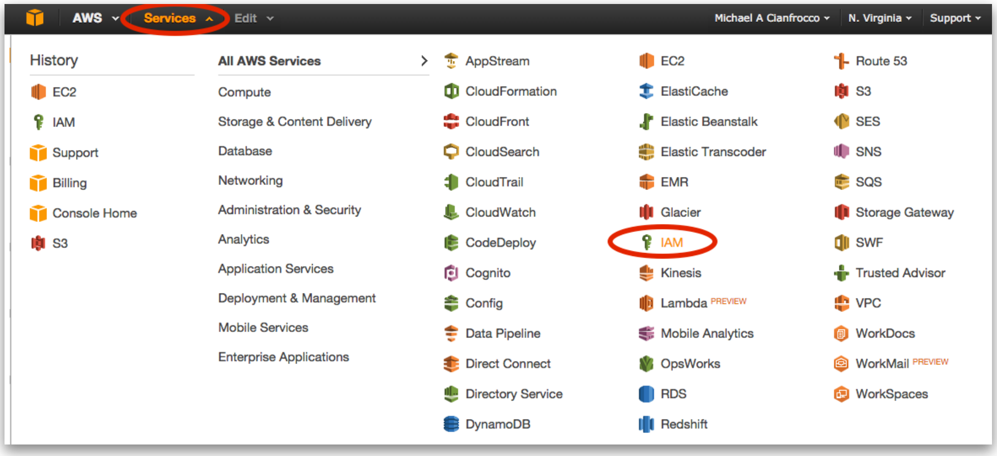

On the next page, go to the '**Activate MFA on your root account**' and click '**Manage MFA**':

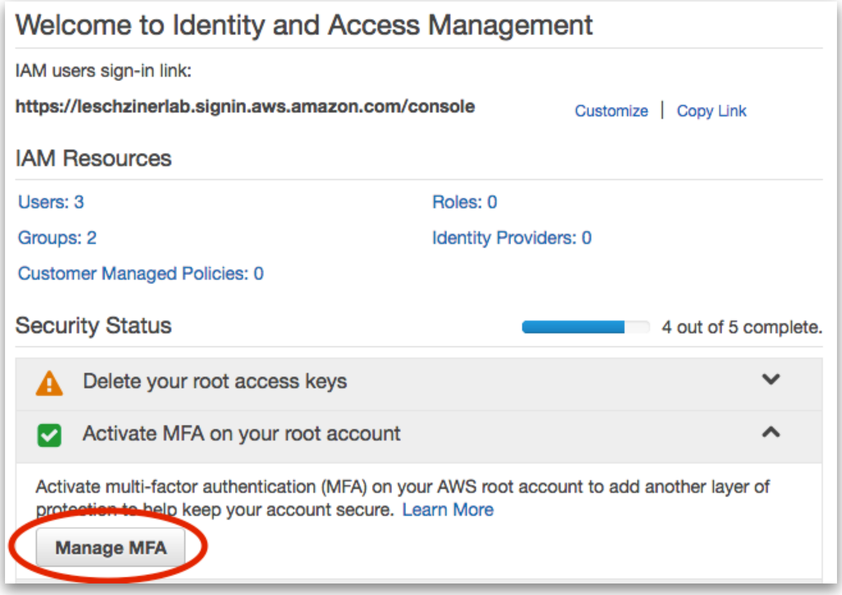

Follow the instructions to set up multi-factor authentication using a virtual device (a smart phone) with Google Authenticator. After setting this up, you will need your smart phone every time you log into AWS as root so that you can enter the code displayed in Google Authenticator.

2. Create new user & give this user 'PowerUser' privileges

Within the IAM interface, you should now create a new user account that YOU will use for day-to-day access / utilization of AWS for data processing.

Navigate to the 'Users' tab on the left-hand side of the IAM interface:

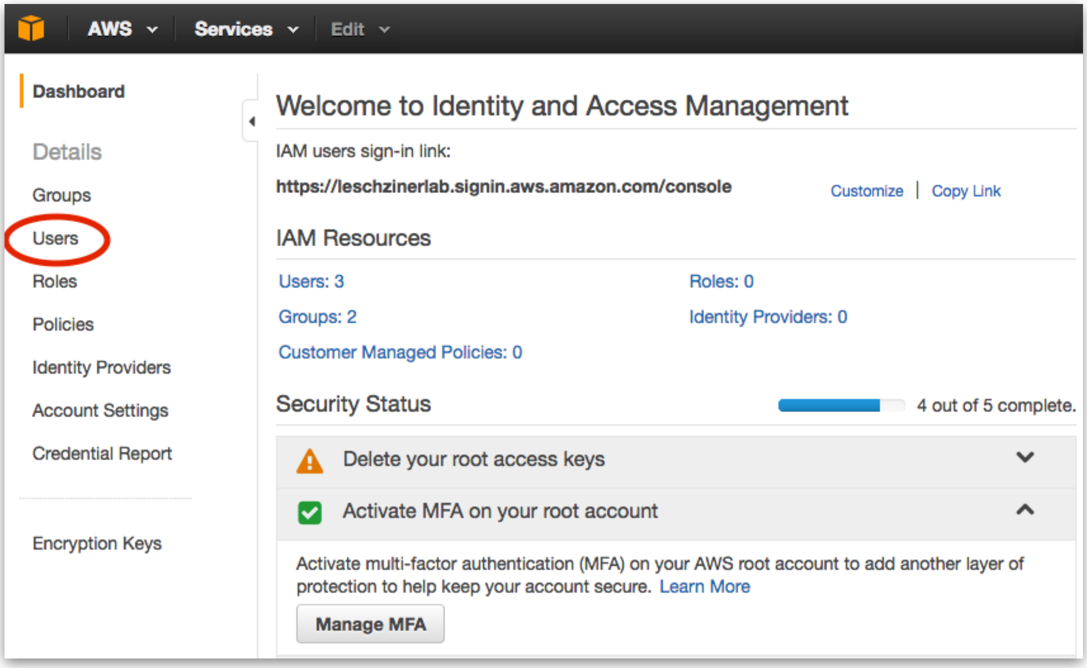

On the next pages:
- Create a new user for yourself
- Download the security credentials (access key & secret ID)
- Create a custom password for this account

Now go to the 'Groups' tab on the left-hand side:
- Create new group
- Name it 'Administrator'
- Select **PowerUser** from the list of choices

After the group is created, go to Group Actions -> Add users to group and select your previously created user name. The log in page is displayed at the IAM homepage. In the above example: https://leschzinerlab.signin.aws.amazon.com/console

Save this and use it for your new user account log in.

3. Set up multi-factor authentication for user

Now that you've set up this new user, you should set up multi-factor authentication for logging in. To do this:

- Go to the users tab while you are still logged in as root
- Select the newly created user name
- Scroll down to the bottom, and then select 'Manage MFA device'
- Follow instructions to set up MFA for this user

4. (Optional) Repeat for users from your lab

Now that you've set up your own user account, you can repeat this for any other users from your own lab. This will make sure that the log in credentials are secure as more users start to use AWS within your research group.

Setting up a single machine

Launching a single instance with EM-packages-in-the-cloud

After setting up your account, to run commands on a single machine, you can follow these steps.

FIRST, make sure you **select an Amazon region** that is close your physical location, it will speed up data transfer. This can be done by selecting it from the top right menu:

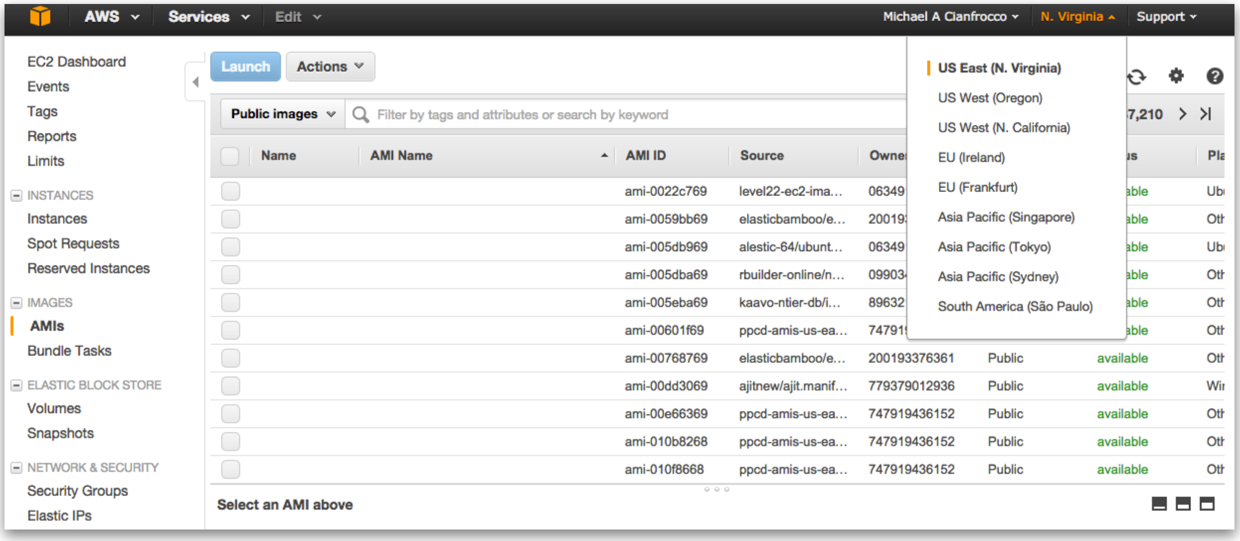

To launch an instance, navigate to the EC2 Management console (from the AWS login screen) and click 'AMIs':

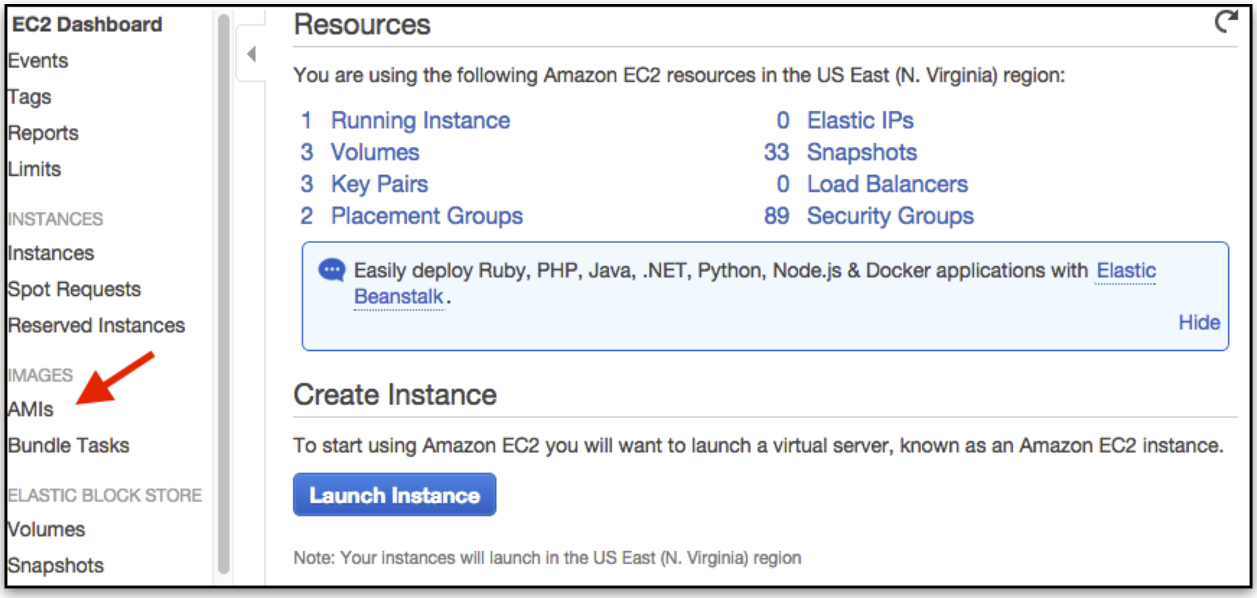

On the next screen, you need to search for **public** AMIs. To do this, first make sure you are displaying 'Public Images', which can be selected by changing the filter by clicking on 'Owned by me' and selecting 'Public Images':

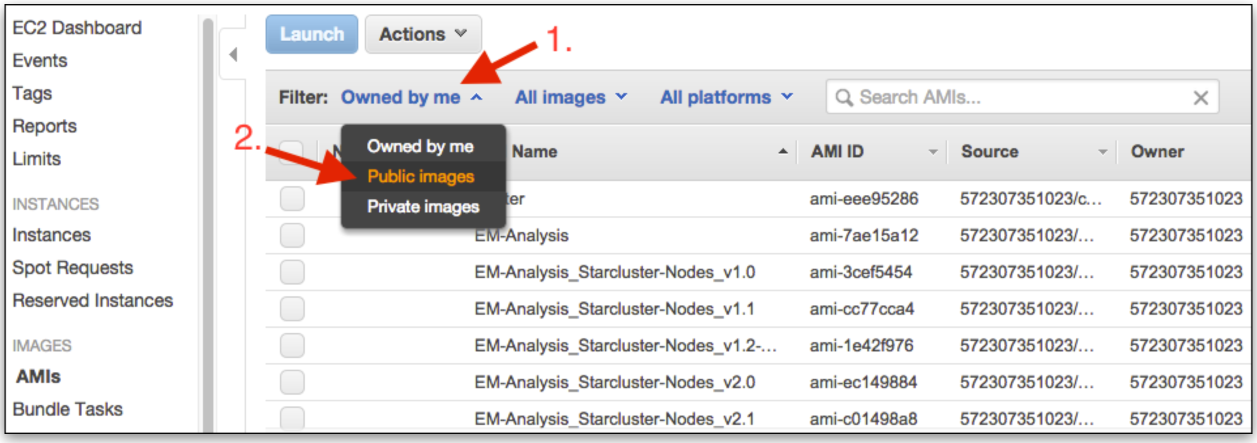

Then search for the name '**EM-packages-in-the-Cloud**', select it, and click '**Launch**': (NOTE: Do not select the other environment that is specific for the cluster nodes, which is named EM-packages-in-the-Cloud-v3.0-node):

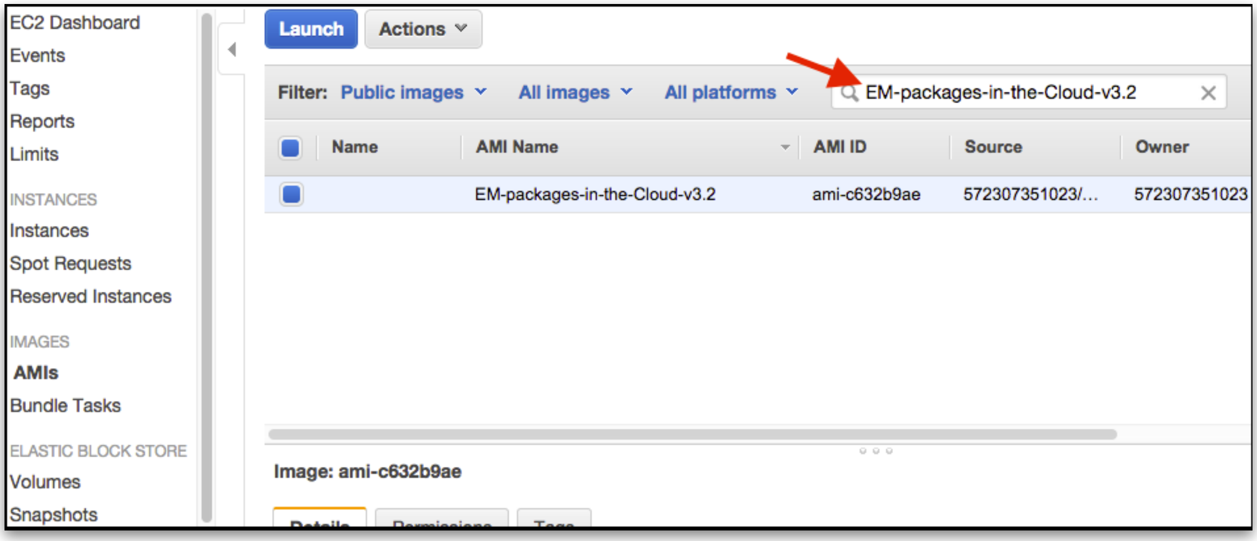

Then select the type of instance that you would like to use. Typically, if we are running our jobs on a single instance, we use compute optimized ('C3'). Otherwise if we are launching this instance to start up a STARcluster, then we select t2.micro:

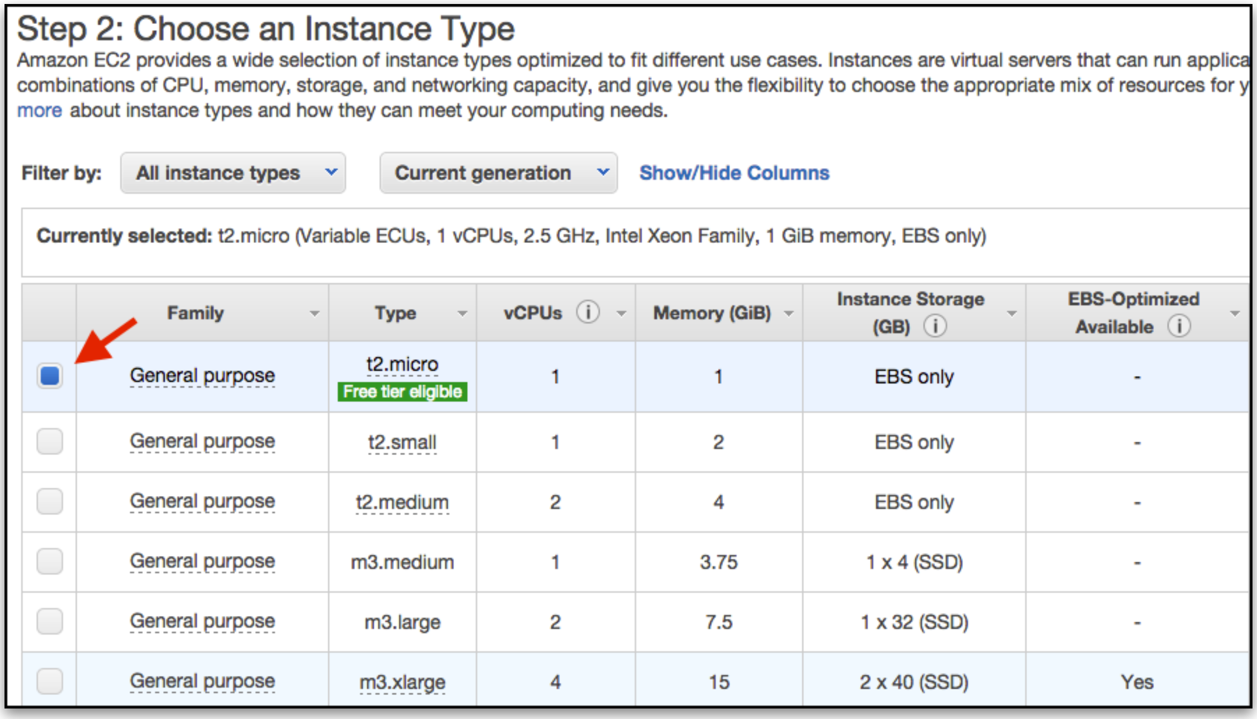

Select '**Next: Configure Instance Details**'.

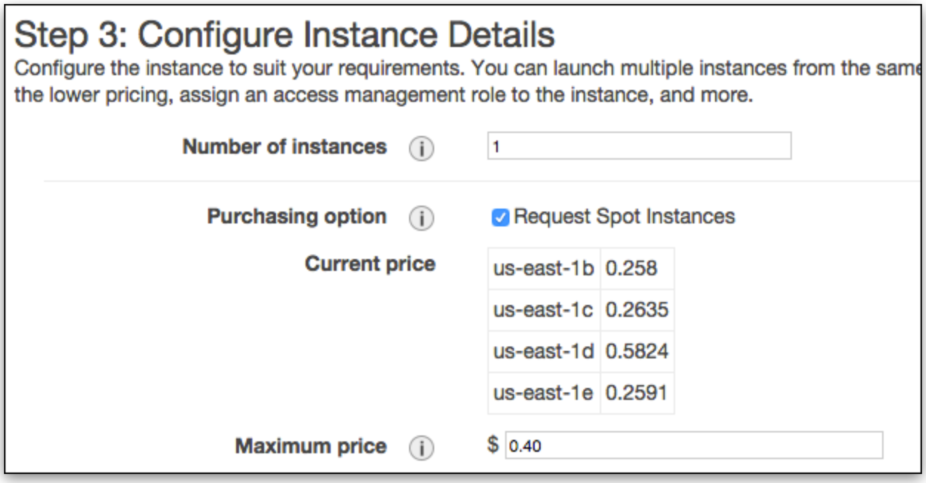

IF you are using the free tier:

- Select subnet that will be used to attached to your volume
    - If you've already created an EBS volume, find out onto which subnet the volume the found, and request this subnet
- Click 'Review and Launch'. It will open a new window asking for a key pair.
    - If this is your first time, create a key pair and save it to your computer, and then select the keypair from the drop down menu

IF you are NOT using the free tier select:

- Request Spot Instance:
    - Input bid for spot instance request (see below for more info on spot instance requests)
- Select subnet that will be used to attached to your volume
    - If you've already created an EBS volume, find out onto which subnet the volume the found, and request this subnet
- Click 'Review and Launch'. It will open a new window asking for a key pair.
    - If this is your first time, create a key pair and save it to your computer, and then select the keypair from the drop down menu (see above)

On the next page, you can utilize **security groups** to help make your Amazon instance more secure. For instance, we always select **Use my IP address**' so that access to your instance is restricted to only your IP address.

Click on Edit security groups:

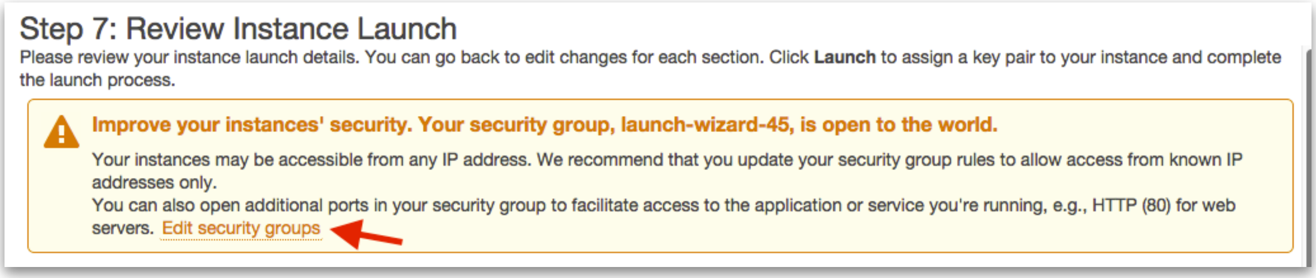

Then, on the next page, select My IP Address so that only users from your IP address can log onto your instance, and then select Review and Launch:

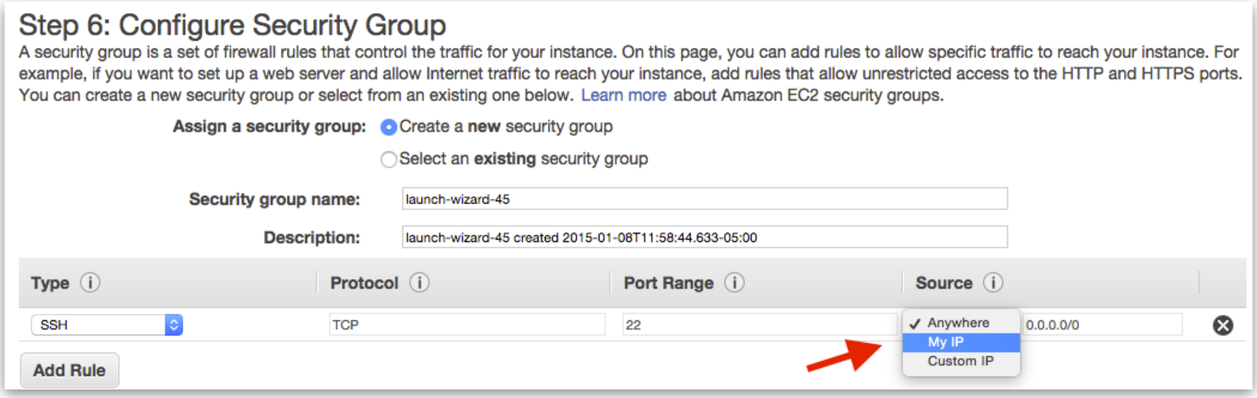

On the Review and Launch page, click Launch. This will bring up a window for you to select your keypairs:

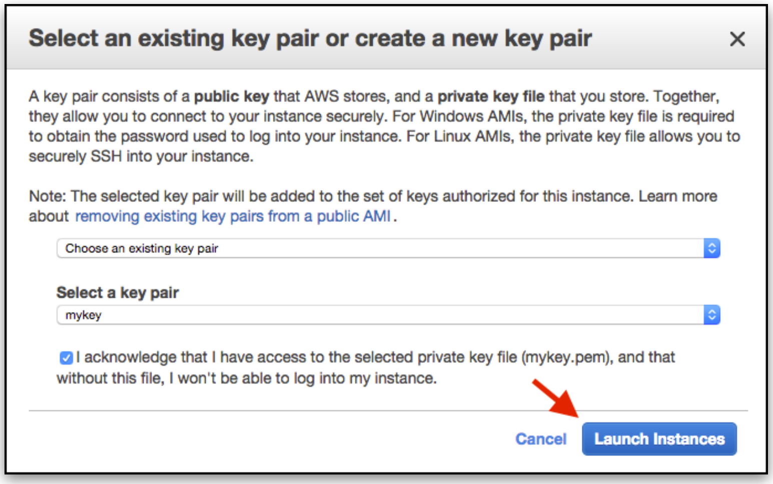

Now you can watch the instance boot up on EC2 Management console page. Once it is booted up, it will display the public IP address that you will use to log into the instance:

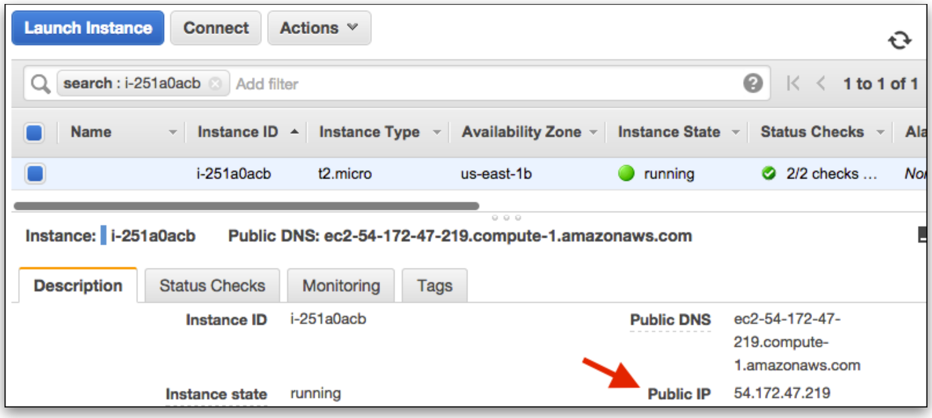

Using the IP address you can log into the instance using ssh.

- If this is your first time downloading the key pair, change the permissions of your keypair:

```
$ chmod 600 mykey.pem
```

Now you can log onto your instance from the command line:

```
$ ssh -X -i mykey.pem ubuntu@54.172.47.219
```

Spot instance requests

Instead of a dedicated instance at full price, Amazon allows its clients to bid on unused instances above a minimum amount. This will let you save 20 - 60% on the price of instances. The only catch, however, is that if someone outbids your price, you will be kicked off of the instance. For example, the r3.8xlarge instance is normally $2.80 when you reserve it as an on-demand instance.

While launching the instance through the web interface, you will have the choice to request a spot instance. By checking 'Request Spot Instances', you will be shown the current price for spot instances. In order to secure a spot instance, you just need to place a bid that is higher than the current price. Minimally, this can be 1 cent over the current price, but practically it is better to do it 5 - 20 cents (or more) to ensure that you won't get kicked off.

EM-Packages-in-the-Cloud AMI

We have set up an AMI that allows users to launch Amazon instances with EM software packages that have been pre-compiled and are ready to use once the instance boots up.

The latest release of EM-Packages-in-the-Cloud-3.92 has the following programs installed:

```
Relion-1.3
EMAN2-2.1
Sparx
EMAN1-1.9
Frealign-9.08
Xmipp-2.4
ctffind3 & ctftilt
Signature
Spider-22.03
TiltPicker
```

If there is a software package or version that you want, just download EM-Packages-in-the-Cloud and install the software, share it publicly, and let everyone know on the Help Forum!

Running commands

All software commands have been put into the $PATH of the shell, so after logging into the instance you can run commands (e.g. *relion_refine_mpi, e2proc2d.py, xmipp_normalize, frealign, spider*)

Data storage

After booting up an instance, any data that you put onto this instance will be deleted once the instance is turned off. Therefore, to create a data storage drive that will persist between instances, we upload our data onto EBS (elastic block storage) drives. EBS drives provide cost-effective storage ($0.10 / GB / month) that can host your data while running calculations on the instances.

This is the overall process:

1. Create a volume on the Amazon EC2 management console
2. Attach volume to an instance
3. Mount volume on instance
4. Upload data
5. Creating a new EBS drive

Create a volume on the Amazon EC2 management console

Navigate to the Elastic Block Storage 'Volumes' tab and select 'Create Volume':

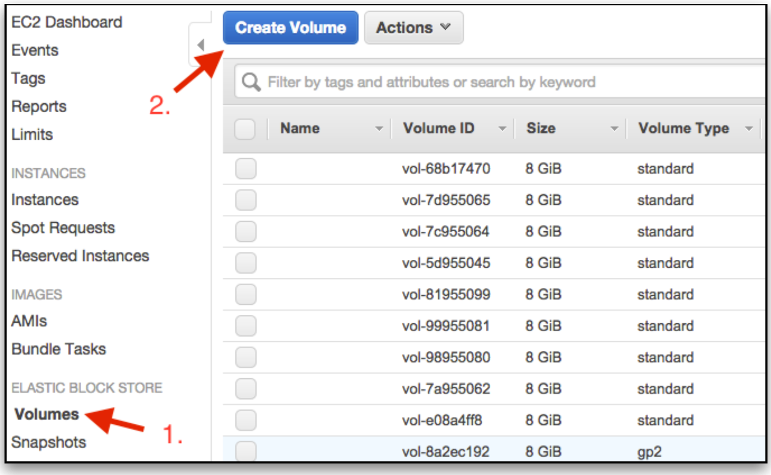

Then, on the next window, enter the size of the volume and whether you want it encrypted:

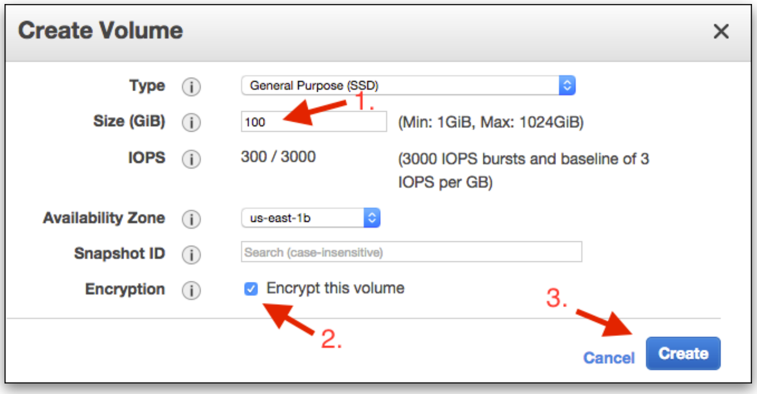

Attaching a volume to an instance

Before attaching a volume, you first need to start an instance (p. 5) to which it will be attached.

Now that the volume has been created and you are running an instance, attach the volume to your instance by first selecting the volume that you want to attach, and then clicking **Actions > Attach Volume**. You will need to input the instance that is running (the available instances within the region will automatically pop up).

Mounting a volume

Now that you have attached the volume to the running instance, you need to mount the volume.

First, you should be able to see the drive on your instance ('xvdf'):

```
$ lsblk
NAME MAJ:MIN RM SIZE RO TYPE MOUNTPOINT
xvda 202:0 0 8G 0 disk
 └──xvda1 202:1 0 8G 0 part /
xvdf 202:80 0 100G 0 disk
```

If this is a new volume (i.e. you just created it), you should see that there is no filesystem on this drive by seeing the following output:

```
$ sudo file -s /dev/xvdf
/dev/xvdf: data
```

Otherwise, if there is a filesystem, it will say something like:

```
$ /dev/xvdf: Linux rev 1.0 ext4 filesystem data, UUID=6c3bf33d-c9bb-4476-
b747-370035489295 (extents) (large files) (huge files)
```

If there is no filesystem, you need to create a new file system by typing:

```
$ sudo mkfs -t ext4 /dev/xvdf
```

But if you are mounting an existing volume, skip the mkfs step and proceed to the next step.

Now create a folder where the volume will be mounted (e.g. /data), and mount the volume:

```
$ sudo mkdir /data
$ sudo mount /dev/xvdf /data
```

Note that we specified /dev/xvdf, which is the drive that was listed when you typed lsblk
There are other volume names that could exist (e.g. xvdg)

Upload your data

Now you can transfer your data to the mounted volume using scp or rsync. We typically get ~25 MB/s upload, which is manageable for small datasets.

Unmounting a volume

When you are finished, you need to unmount the volume:

```
$ sudo umount -d /dev/xvdf
```

Then detach the volume on the Amazon EC2 management console by selecting the volume, and then **Actions > Detach Volume**.

Setting up a cluster

Cluster setup workflow:

1. Start an Amazon EC2 instance
    - We start the t2.micro because it is free
2. Mount and upload data onto an EBS volume
    - Unmount EBS volume when finished uploading data
3. Edit .starcluster/config file
    - Input security credentials
    - Input EBS volume information
4. Create keypair for starcluster
5. Check current spot request minimum bid
6. Start starcluster

Configuring your cluster through .starcluster/config

The following assumes that you already have started an Amazon instance and that you are editing the .starcluster/config file. This file is located in your home directory:

`/home/ubuntu/.starcluster/config`

The configuration file for starcluster contains A LOT of information that can be used to specify how your cluster is set up. You'll notice that some of it is commented out with a '#' symbol. The following steps will direct you towards the most pertinent settings that you need to input.

Gathering security credentials

How to find the security credentials:

`AWS_USER_ID`

- Navigate to **Pull down menu with your name on it** (top right corner of EC2 Management console)
- Click **My Account**
- Your user ID will be on the next page next **Account Id**

`AWS_ACCESS_KEY_ID`

- Navigate to **Pull down menu with your name on it** (top right corner of EC2 Management console)
- Click **Security credentials**
- Click **Continue to security credentials**
- Select **Access Keys menu**
- Create **New Access Key**, you need to create a new access key.
- You'll see the **Access Key displayed**
- **Download** access key for later reference

`AWS_SECRET_ACCESS_KEY`

- After you create a New Access Key, either display the secret key by clicking on the tab in the 'New Access Window'
    - Or download the secret key to your computer
    - Keep in mind that this is the only time you'd have access to the Secret Access Key, so you'll have to copy it down or save it
    - You can always create a New Access Key whenever you want, at which point you'd receive a new Secret Access Key.

Editing .starcluster/config

Now open the .starcluster/config file and enter your own user information for the following lines:

```
AWS_USER_ID=

AWS_ACCESS_KEY_ID =

AWS_SECRET_ACCESS_KEY =

###################

VOLUME_ID =                  <----- Specify volume ID name (e.g. vol-fe1aa3ab)
MOUNT_PATH = /data                   <----- Path where volume will be mounted

###################

CLUSTER_SIZE = 1                     <----- Specify cluster size here
NODE_INSTANCE_TYPE = r3.8xlarge      <----- Specify instance type
```

Create keypair

Then you need to **create a keypair** for your master node and slave nodes. To create a new keypair:

```
$ starcluster createkey mykey -o ~/.ssh/mykey.rsa

>>> Successfully created keypair: mykey

>>> fingerprint: [keypair fingerprint here]

>>> keypair written to /home/ubuntu/.ssh/mykey.rsa
```

It needs to be named **mykey.rsa** and placed into this location.

If you receive any error about the keypair already existing, simply make sure any file with your your keyname.rsa is removed from .ssh/ and then run the command:

```
$ starcluster removekey mykey
```

And then try to create the keypair again.

On-demand vs. spot instances

Now that you are ready to start your cluster, you need to decide if you are going to use on-demand instances or spot instances. On-demand instances are priced according the prices

listed here. The benefits of on-demand instances is that you cannot get kicked off of your instance (unlike spot instances). For this privilege you will pay a higher rate per hour.

Instead of paying the full price for the instances, you can request 'spot instances'. These are unused instances that Amazon puts up to auction - users bid to use these instances and compete for their use.

To find out the current rate for a spot instance:

```
$ starcluster spothistory r3.8xlarge

>>> Fetching spot history for r3.8xlarge (VPC)

>>> Current price: $0.2573

>>> Max price: $2.8000

>>> Average price: $0.4899
```

For this example, the current price for a spot instance is $0.2573, which is less than 10% of the the on-demand rate for r3.8xlarge ($2.80)! The only negative aspect of this approach is that you can get kicked off of your instance at any time if someone outbids you. But, that said, it doesn't seem to happen very often and your data are always saved on your EBS.

Starting a STARcluster

Now you are ready to go! To start it up using spot instances:

```
$ starcluster start mycluster --bid=0.40 --force-spot-master
```

Where 'mycluster' is the name provided for your cluster, the bid price is greater than the current price, and '—force-spot-master' ensures that the master node is a spot instance. 'mycluster' will be used throughout this tutorial as the cluster name.

Alternatively, if you want to pay the FULL price for the instances, you would enter:

```
$ starcluster start mycluster
```

Logging into the cluster

To log into your cluster:

```
$ starcluster sshmaster mycluster -X -u cluster_user
```

Where -X specifies X11 window forwarding and -u specifies the user.

Important: The environment paths to the software packages are set up for the user 'cluster_user', so you need to log into the cluster using this username.

To log in as root, don't specify cluster_user:

```
$ starcluster sshmaster mycluster -X
```

Example job submission script

Here is an example submission script for running a 3D classification routine within Relion:

```
#!/bin/bash
#$ -cwd                 #Run from current working directory
#$ -S /bin/bash         #Shell
#$ -pe orte 64          #Number of CPUs to use
#$ -q all.q             #Queue type. Default = all.q
#$ -V                   #Specify same running environment
#$ -N r3_8xlarge_2      #Name of job

mpirun relion_refine_mpi --o Class3D/run_r3_8xlarge_2 --i particles_sel.star
--particle_diameter 350 --angpix 3.54 --ref ref_filt60A_sca.mrc --firstiter_cc
--ctf --iter 2 --tau2_fudge 2 --K 4 --flatten_solvent --zero_mask --
oversampling 1 --healpix_order 3 --offset_range 5 --offset_step 2 --sym C1 --
norm --scale  --j 1 --memory_per_thread 10 --dont_combine_weights_via_disc
```

IMPORTANT: Specifying —dont_combine_weights_via_disc for both Relion 3D classification and refinement dramatically speeds up (10X) the calculations

And then to submit the script:

```
$ qsub submission_script.run
```

You will see command line outputs to the standard out file (e.g. r3_8xlarge_2.o1) and errors to a separate file (e.g. r3_8xlarge_2.e1).

Useful cluster commands

qstat
Lists jobs that are in the queue

qstat -g c
Displays the number of total, available, and busy CPUs within cluster

qstat -f
Shows a list of nodes along with total number of CPUs and number of CPUs being used

qsub

Used to submit jobs to the cluster (e.g. $ qsub submit.csh)

qdel
Delete job from cluster using the job number (e.g. $ qdel 2)

Stopping cluster

To automatically stop a cluster when a job finishes, you can use the Elastic Load Balancer feature. After starting a cluster and submitting it to the STARcluster, log out of the cluster and type:

```
$ starcluster loadbalance --kill-cluster mycluster
```

Alternatively, you can manually shut down a cluster:

```
$ starcluster terminate mycluster -f
```

Troubleshooting

If you run into any issues, we have created a Google Group help forum **Cryo-EM in the cloud** where users can share solutions to any problems that they encounter:

https://groups.google.com/forum/#!forum/cryo-em-in-the-cloud

Also, be sure to check out our website for latest news and updates:

https://sites.google.com/site/emcloudprocessing/
