# Supplementary material for: Low cost, high performance processing of single particle cryo-electron microscopy data in the cloud
Source: eLife. 2015 May 8;4:e06664. doi: 10.7554/eLife.06664 (PMC4440898; doi:10.7554/eLife.06664)
Supplement: Supplementary file 2. — Comparison of estimated processing times and costs for recent near-atomic cryo-EM structures on Amazon's EC2. Source data: Supplementary File 3. DOI: http://dx.doi.org/10.7554/eLife.06664.011 [file elife06664s004.docx]

**Supplemental File 2: Comparison of estimated processing times and costs for recent near-atomic cryo-EM structures on Amazon’s EC2.**

**Supplemental File 2A: Overview of 3D refinement processing times for recent near-atomic cryo-EM structures.**

| **3D refinement processing times and estimated costs on Amazon EC2**^§^ | | | | | | |
| --- | --- | --- | --- | --- | --- | --- |
|  |  |  | **EC2 cost at specified spot instance prices** | | | |
|  | **Resolution** | **CPU Hours** | **$0.35** | **$0.45** | **$0.55** | **$0.65** |
| **80S eukaryotic ribosome^#^** | 4.6 Å | 578 | $12.65^^^ | $16.26^^^ | $19.87^^^ | $23.48^^^ |
| **γ-secretase^¶^** | 4.5 Å | 4,660 | $101.94^*^ | $131.06^*^ | $160.19^*^ | $189.31^*^ |
| **β-galactosidase^‡^** | 4.0 Å | 1,160 | $25.38^*^ | $32.63^*^ | $39.88^*^ | $47.13^*^ |
| **Yeast mitochondrial ribosome^†^** | 3.3 Å | 9,330 | $204.09^*^ | $262.41^*^ | $320.72^*^ | $379.03^*^ |

§ Adapted from (Scheres, 2014)

# Our test sample. 3D refinement time for re-analysis of 80S ribosome data from (Bai et al., 2013) to show processing time on Amazon’s EC2 infrastructure

¶ (Lu et al., 2014)

‡ (Scheres and Chen, 2012)

† (Amunts et al., 2014)

^ Actual cost on Amazon’s EC2 infrastructure

* Predicted cost based on 128 CPUs (STARcluster of eight r3.8xlarge instances)

**Supplemental File 2B: Overall processing times and associated costs for recent near-atomic cryo-EM structures.**

| **Estimated overall processing time & costs on Amazon EC2** | | | | | | |
| --- | --- | --- | --- | --- | --- | --- |
|  |  |  | **EC2 cost at specified spot instance prices** | | | |
|  | **Resolution** | **Total CPU Hours** | **$0.35** | **$0.45** | **$0.55** | **$0.65** |
| **80S eukaryotic ribosome^#^** | 4.6 Å | 1,266 | $54.38 | $69.68 | $84.66 | $99.64 |
| **γ-secretase^¶^** | 4.5 Å | 9,430 | $409.96^*^ | $526.44^*^ | $642.96^*^ | $759.44^*^ |
| **β-galactosidase^‡^** | 4.0 Å | 2,430 | $103.72^*^ | $132.72^*^ | $161.72^*^ | $190.72^*^ |
| **Yeast mitochondrial ribosome^†^** | 3.3 Å | 18,770 | $818.56^*^ | $1,051.84^*^ | $1,282.20^*^ | $1,518.32^*^ |

# Total CPU hours and cost for re-analysis of 80S ribosome data from (Bai et al., 2013)

¶ (Lu et al., 2014)

‡ (Scheres and Chen, 2012)

† (Amunts et al., 2014)

* Predicted cost was based on 110 hours at $0.02/hr on m1.small instance ($2.20) to pick particles, estimate CTF, and extract particles. To estimate 2D and 3D analyses, we assumed that 3D classification required an equal number of CPU hours as 3D refinement, and 2D classification required an equal number of CPU hours as both 3D classification and 3D refinement: Estimated cost = $2.20 + 4*(3D refinement cost). The estimation also assumed that the 2D & 3D analyses were calculated on a 128 CPU STARcluster of eight r3.8xlarge instances.
